# Reelin-Haploinsufficiency Disrupts the Developmental Trajectory of the E/I Balance in the Prefrontal Cortex

**DOI:** 10.3389/fncel.2016.00308

**Published:** 2017-01-12

**Authors:** Lamine Bouamrane, Andrew F. Scheyer, Olivier Lassalle, Jillian Iafrati, Aurore Thomazeau, Pascale Chavis

**Affiliations:** INMED, Aix-Marseille University, INSERMMarseille, France

**Keywords:** prefrontal cortex, GABA, reelin, postnatal maturation, synaptic transmission, E/I balance

## Abstract

The reelin gene is a strong candidate in the etiology of several psychiatric disorders such as schizophrenia, major depression, bipolar disorders, and autism spectrum disorders. Most of these diseases are accompanied by cognitive and executive-function deficits associated with prefrontal dysfunctions. Mammalian prefrontal cortex (PFC) development is characterized by a protracted postnatal maturation constituting a period of enhanced vulnerability to psychiatric insults. The identification of the molecular components underlying this prolonged postnatal development is necessary to understand the synaptic properties of defective circuits participating in these psychiatric disorders. We have recently shown that reelin plays a key role in the maturation of glutamatergic functions in the postnatal PFC, but no data are available regarding the GABAergic circuits. Here, we undertook a cross-sectional analysis of GABAergic function in deep layer pyramidal neurons of the medial PFC of wild-type and haploinsufficient heterozygous reeler mice. Using electrophysiological approaches, we showed that decreased reelin levels impair the maturation of GABAergic synaptic transmission without affecting the inhibitory nature of GABA. This phenotype consequently impacted the developmental sequence of the synaptic excitation/inhibition (E/I) balance. These data indicate that reelin is necessary for the correct maturation and refinement of GABAergic synaptic circuits in the postnatal PFC and therefore provide a mechanism for altered E/I balance of prefrontal circuits associated with psychiatric disorders.

## Introduction

Reelin is a signaling glycoprotein (Bock and May, 2016) serving multiple functions in the brain throughout life which has also emerged as a psychiatric risk factor in a wide spectrum of psychiatric disorders (Folsom and Fatemi, 2013). Secreted by Cajal-Retzius cells in the marginal zone of the cerebral cortex and hippocampus and by pioneer granule cells of the cerebellum during embryonic development, reelin plays an essential role in neuronal migration, positioning and layer formation (Sekine et al., 2014). In addition to being a developmental molecule, reelin is an important contributor to postnatal and adult central nervous system (CNS) physiology. In postnatal forebrain, once migration and layering are completed, reelin production is shifted to subpopulations of GABAergic interneurons distributed throughout cellular layers of the hippocampus and neocortex (Alcantara et al., 1998; Pesold et al., 1998; Campo et al., 2009). In the maturing and adult CNS, reelin modulates several aspects of excitatory synaptic function and morpho-functional plasticity. Reelin plays an important role in dendritic maturation and spine development (Niu et al., 2008; Chameau et al., 2009), hippocampal long-term potentiation, synaptic transmission, and cognitive ability (Weeber et al., 2002; Beffert et al., 2005; Pujadas et al., 2010; Rogers et al., 2011). Additionally, previous data from our laboratory have shown that reelin is necessary for the maturation of NMDA receptors (Sinagra et al., 2005; Groc et al., 2007; Campo et al., 2009). More recently, we have shown that spine density, excitatory synaptic transmission and plasticity of prefrontal pyramidal neurons as well as cognitive traits are altered during the postnatal maturation of the reelin-haploinsufficient heterozygous reeler mice (HRM) prefrontal cortex (PFC) (Iafrati et al., 2014; Iafrati et al., 2016). We showed that reelin is necessary for the correct structural and functional maturation of deep layer excitatory synapses of the prelimbic area of the PFC (PrPFC) and that reelin happloinsufficiency delineates prefrontal endophenotypes thus identifying reelin as a risk gene for PFC maturational cognitive deficits (Iafrati et al., 2014; Iafrati et al., 2016). Despite these advancements, and apart from studies reporting alterations in GABAergic markers and reduced number of purkinje cells in HRM (Hadj-Sahraoui et al., 1996; Biamonte et al., 2009; Nullmeier et al., 2011) as well as the correlation between firing properties and neurochemical identity of reelin-expressing interneurons (Pohlkamp et al., 2014), the role of reelin in the maturation and plasticity of GABAergic connectivity has not been investigated.

A significant contribution of reelin to the etiology of psychiatric and neurodevelopmental disorders has been proposed based on evidences of the pleiotropic roles of reelin in adult and developing brain together with patients’ data showing alteration in reelin levels (Folsom and Fatemi, 2013). Patients suffering from psychiatric disorders such as schizophrenia, bipolar disorder, major depression, and autism spectrum disorders (ASDs) exhibit an approximate reelin downregulation of 50% in several brain structures, most notably the hippocampus and PFC (Impagnatiello et al., 1998; Guidotti et al., 2000; Folsom and Fatemi, 2013). In schizophrenic patients, reduced reelin levels were accompanied by the decrease of other interneurons markers (Impagnatiello et al., 1998; Guidotti et al., 2000; Fatemi et al., 2005). As such, the identification of the mechanisms by which reelin contributes to GABAergic circuit dysfunctions in these diseases is of considerable interest.

The PFC is an associative brain region that supports complex cognitive functions. In rodents, the prelimbic area is one of the regions of the medial PFC which likely mediates cognitive functions similarly to the primate dorsolateral PFC (Kesner and Churchwell, 2011). One distinctive feature of the PFC is its protracted maturation through early adulthood (Gogtay et al., 2004) characterized by connectivity refinement in parallel to maturation of cognitive abilities (van Eden et al., 1990; Luna et al., 2001). This extended period of maturation constitutes a sensitive period of increased vulnerability to injuries leading to development of neurophychiatric disorders (Lewis, 1997; McEwen and Morrison, 2013; Iafrati et al., 2016; Labouesse et al., 2016). Several studies suggest that alterations of postnatal PFC maturation may contribute to the development of psychiatric diseases including depression, addiction, ASD and schizophrenia (Lewis, 1997; Raedler et al., 1998; Iafrati et al., 2016). Neuronal deficits associated to these disorders could include reduced elaboration of inhibitory connectivity leading to altered excitation–inhibition (E/I) balance in the PFC (Insel, 2010). Indeed, a general reduction of the GABAergic system has been described in the PFC of schizophrenic (Volk et al., 2000; Guidotti et al., 2005; Torrey et al., 2005; Gonzalez-Burgos and Lewis, 2008) autistic (Fatemi et al., 2002; Fatemi et al., 2010; Oblak et al., 2011; Fatemi et al., 2014) and depressive patients (Sanacora et al., 1999, 2004; Hasler et al., 2007; Bhagwagar et al., 2008; Karolewicz et al., 2010). Down-regulation of the GABAergic system is also reported in several animal models of psychiatric diseases (Beninger et al., 2010; Cellot and Cherubini, 2014). However, it is not known whether prefrontal GABAergic function and connectivity is affected by reelin haploinsufficiency.

In the present study, we analyzed the polarity of GABAergic signaling, maturation of GABAergic synaptic transmission and the E/I balance in deep layer PrPFC pyramidal neurons of wild-type mice and HRM throughout the first 3 months of postnatal life. We provide evidence that reelin is crucial for correct maturation of GABAergic synaptic functions and E/I balance in the postnatal PFC.

## Materials and Methods

### Animals

The HRM (B6C3Fe a/a-Relnrl/J strain) breeding pairs were purchased from Jackson Laboratory. Offsprings were genotyped by PCR as previously described (Iafrati et al., 2014). Males and females were used in electrophysiological and morphological studies and no significant sex-dependent differences were observed. All mice were weaned at 21 days and then caged socially in same-sex groups. Mice were housed in standard 12 h light–dark cycle and supplied food pellets and water *ad libitum*. Animals were treated in strict compliance with the criteria of the European Communities Council Directive (agreement number 2015121715284829-V4).

### Electrophysiology

Coronal slices containing the prelimbic area of the medial prefrontal cortex (PrPFC) were prepared as previously described (Lafourcade et al., 2007). Briefly, mice were anesthetized with isoflurane and 300 μm-thick coronal slices were prepared in a sucrose-based solutiuon at 4°C using an Integraslice vibratome (Campden Instruments). Slices were stored for 30 min at 32°C in artificial cerebrospinal fluid (ACSF) containing 130 mM NaCl, 2.5 mM KCl, 2.4 mM MgCl_2_, 1.2 mM CaCl_2_, 23 mM NaHCO_3_, 1.2 mM NaH_2_PO_4_ and 11 mM glucose, equilibrated with 95% O_2_/5% CO_2_. Slices were then stored at room temperature until recording. All experiments were conducted at 30–32°C in ACSF. Whole-cell and cell-attached patch-clamp recordings were made in PrPFC layer 5/6, collected using an Axopatch-1D amplifier (Axon Instruments) and acquired with Clampex 10.2 acquisition Software via a Digidata 1440A (Axon Instruments). Pyramidal neurons in PrPFC layer 5/6 were visualized using an infrared illuminated upright microscope (Olympus BX51WI).

#### Spontaneous Spiking Activity

Spontaneous spiking activity was recorded in cell-attached configuration with a patch pipette filled with ACSF. A gigaOhm seal was obtained in current clamp configuration before recording spike-activity in *I* = 0 mode. Data were filtered at 2 kHz and digitized at 10 kHz. Spontaneous spike activity was analyzed in Clampfit 10.5 (Molecular Devices) threshold detection with a trigger threshold of >2x SD of baseline noise. Mean spike activity was calculated as an average of spikes per minute over a 10-min baseline period. For drug-effects, mean spike activity was calculated as an average of spikes per minute over a 10-min period following at least 5 min of bath perfusion.

#### Spontaneous and Evoked GABA-IPSCs, Spontaneous AMPA-EPSCs, and Intrinsic Properties

To record GABA_A_-mediated PSCs, NBQX (10 μM) and DL-APV (100 μM) were added to the ACSF to block glutamatergic synaptic transmission. Spontaneous GABA_A_ receptor-mediated inhibitory post-synaptic currents sIPSCs and evoked IPSCs (eIPSCs) were recorded at -70 mV using the following intracellular solution which contained (mM): Cesium-Cl (125), KCl (20), EGTA (1), HEPES (10), Na_2_ATP (2), NaGTP (0.3), and cAMP (0.2) (pH 7.3 and 290 mOsm). In these conditions, chloride reversal potential was around 0 mV. To record spontaneous AMPA-EPSCs (AMPA-sEPSCs) picrotoxin (100 μM; Sigma) was added to the ACSF to block GABA_A_ synaptic transmission. AMPA-sEPSCs were recorded at -70 mV using internal solution containing (mM): K-Gluconate (145), NaCl (5), MgCl_2_ (1), EGTA (1), CaCl_2_ (0.3), Hepes (10), Na_2_ATP (2), NaGTP (0.3), and 0.2 cAMP (0.2) (pH 7.3 and 290 mOsm) (Iafrati et al., 2014). To perform current-voltage curves and test neuronal excitability a series of hyperpolarizing and depolarizing current steps were applied immediately after breaking in the cell.

Whole-cell recording electrodes had resistances of 4–6 MOhms. Access resistance was continuously monitored (<25 MOhms) and recordings were rejected if there was a >20% change during the course of the experiment. Spontaneous and evoked currents were filtered at 2 kHz and digitized at 10 kHz.

Paired-pulse ratio (PPR) was measured from IPSCs evoked by a stimulating glass electrode filled with ACSF placed in layer 2/3. Time intervals between stimulations were 30, 50, 100, 150, 200, 300, and 400 ms.

Spontaneous post-synaptic currents amplitude and inter-interval time were detected and analyzed with Axograph X using a double exponential template: f(t) = exp(-t/rise) + exp(-t/decay). For GABA-sIPSCs, rise = 0.2 ms and decay = 10 ms, and for AMPA-sEPSCs, rise = 0.5 ms and decay = 3 ms. The threshold of amplitude detection was set at 7 pA.

Total charge was calculated by summing the charge transfer of all individual events (sEPSCs or sIPSCs) detected over a 6 min acquisition period for each neuron.

### Statistical Analysis

All values are given as mean ± SEM and statistical significance was set at *P* < 0.05. Statistical analysis was performed with GraphPad Prism 6.0 (GraphPad Software, La Jolla, CA, USA). Two sample comparisons were made with the non-parametric Mann–Whitney test and multiple comparisons were made using a one-way analysis of variance (ANOVA) followed, if significant, by Tukey’s test.

## Results

In order to evaluate the effects of an extracellular neuronal environment impoverished in reelin on the polarity of GABA action, maturation of GABAergic synaptic transmission and the E/I balance of the PrPFC, we compared wild-type and reelin-haploinsufficient HRM during the first 3 months of postnatal life: pre-weaning (Pw, P14–20), juvenile (Juv, P22–28), adolescent (Ado, P30–45), and adulthood (Adu, P50–90). These developmental epochs match our recent report studying the effect of reelin-haploinsufficiency on the maturation of deep layer PrPFC excitatory synapses (Iafrati et al., 2016). Here, we also focused on layer 5/6 pyramidal neurons, one of the main output cells of the PrPFC microcircuit. Layer 5/6 pyramidal neurons were identified as previously described (Thomazeau et al., 2014; Iafrati et al., 2016; Martin et al., 2016) based on their morphology and/or their intrinsic properties (**Supplementary Figure S1**).

### Polarity of GABA Action Is Not Affected by Reelin Haploinsufficiency during the P14–20 Pre-weaning Period

**FIGURE 1:**
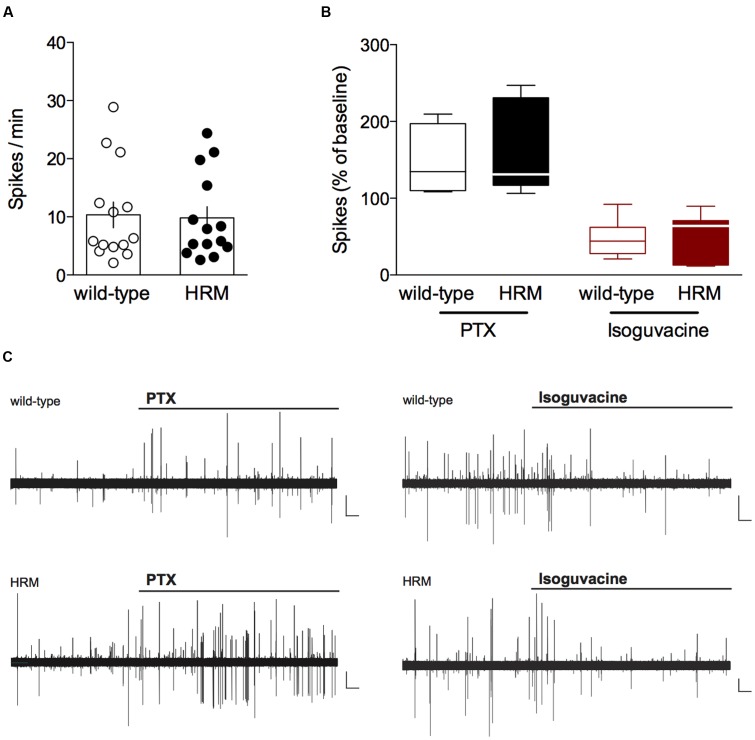
**Inhibitory action of GABA in P14–18 wild-type and reelin-haploinsufficient mice.** **(A)** Average baseline spontaneous spiking activity (number of spikes per min) from cell-attached recorded visually identified deep layer pyramidal neurons from P14-P18 wild-type mice and HRM. Spike frequency was 10.3 ± 2.2 spikes/min (*n* = 14 cells/10 mice) in wild-type mice and 9.8 ± 2.0 spikes/min (*n* = 14 cells/8 mice) in HRM. Data points represent baseline spontaneous spiking frequency from individual cells. Error bars represent SEM. **(B)** Effect of the GABA_A_ antagonist picrotoxin (PTX, 20 μM, black) or the GABA_A_ agonist isoguvacine (7 μM, red) on the spontaneous spiking activity of P14–18 wild-type mice (open symbols) and HRM (filled symbols). Box plot showing the interquartile range with whiskers at minimum and maximum data points of the effect of PTX (wild-type: *n* = 7 neurons/5 mice; HRM: *n* = 7 neurons/4 mice) or isoguvacine (wild-type: *n* = 7 neurons/5 mice; HRM: *n* = 7 neurons/4 mice) expressed as the percentage of baseline spontaneous spiking activity. Horizontal lines represent the median. Note the inhibitory action of PTX and excitatory effect of isoguvacine in both genotypes. **(C)** Representative traces of the excitatory action of PTX and inhibitory effect of isoguvacine on the spontaneous spiking activity recorded in cell attached configuration in layer 5/6 pyramidal neuron from P16 wild-type and HRM. Calibration: 100 pA, 1 s.

One of the first events during postnatal brain maturation is the switch in GABA polarity from depolarization/excitation in immature neurons to hyperpolarization/inhibition in adult neurons (reviewed in (Ben-Ari et al., 2012). Alteration in the timing of this polarity switch has been consensually reported under pathological conditions and in mouse models of ASD and intellectual disability (He et al., 2014; Tyzio et al., 2014; Deidda et al., 2015). Although GABA is depolarizing in immature cortical neurons, it has also been reported to inhibit network activity of the neonatal cortex *in vivo* (Kirmse et al., 2015). In light of these findings, we felt that a prerequisite to studying the maturation of GABAergic synaptic transmission was to examine the polarity of GABA signaling before weaning between P14 and P18 and whether it was altered at this maturational stage in pathological conditions, e.g., in HRM (**Figure 1**).

We tested the direction of GABAergic actions by observing the impact of the GABA_A_ receptor antagonist picrotoxin (PTX) and the GABA_A_ receptor positive-allosteric modulator isoguvacine on cell-attached recorded spontaneous spiking activity of layer 5/6 pyramidal neurons (Khazipov et al., 2004). First, we examined the average baseline spontaneous spiking frequency which was found to be similar in P14–18 HRM and wild-type littermates (*P* = 0.9101, Mann–Whitney *U*-test; **Figure 1A**), suggesting that reelin haploinsufficiency does not affect basal network activity. In P14–18 wild-type mice, application of PTX increased the spontaneous spiking activity to 146.5 ± 15.6% whereas it was reduced to 46.8 ± 9.1% following application of isoguvacine (**Figures 1B,C**). These results demonstrate that in wild-type mice GABA exerts a classical inhibitory action from P14. PTX and isoguvacine produced the same effects on baseline spontaneous activity recorded in P14–18 HRM (increase to 155.5 ± 22.0% and reduction to 53.4 ± 11.3% respectively; **Figures 1B,C**).

Together, these data show that from the pre-weaning P14–18 period GABA exhibits an inhibitory action on layer 5/6 pyramidal neurons in the PrPFC, and that reelin haploinsufficiency does not impact local network activity nor the inhibitory action of GABA during the pre-weaning period.

### Reelin Haploinsufficiency Disrupts the Maturation of GABAergic Synaptic Transmission in Layer 5/6 PrPFC

**FIGURE 2:**
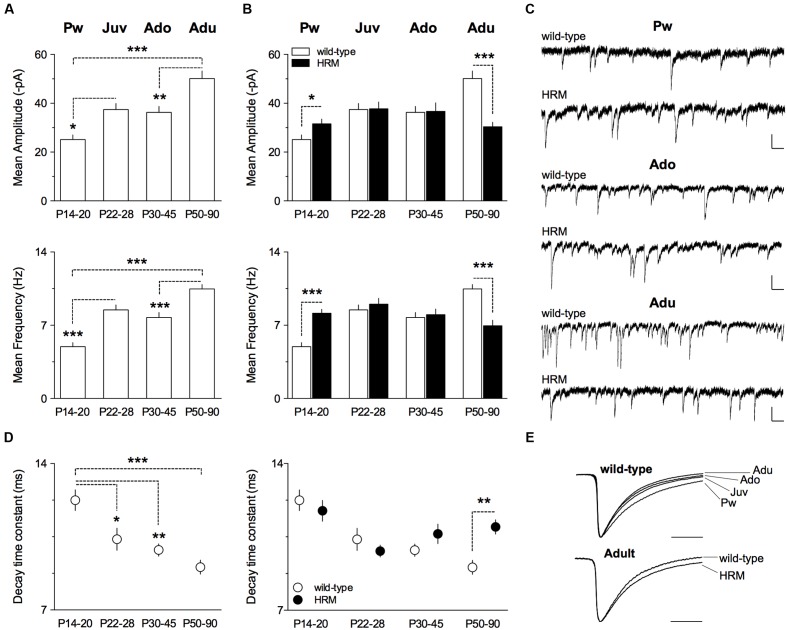
**Maturational profile of GABAergic spontaneous activity in wild-type mice and HRM.** **(A)** Mean amplitude and frequency of spontaneous GABA-IPSCs in wild-type mice from pre-weaning to adult stage. Values for mean amplitude were: 25.1 ± 1.9 pA (*n* = 10 neurons/5 mice) at P14–20, 37.4 ± 2.5 pA (*n* = 13 neurons/9 mice) at P22–28, 36.3 ± 2.5 pA (*n* = 12 neurons/6 mice) at P30–45 and 50.1 ± 3.1 pA (*n* = 16 neurons/7 mice) at P50–90. Values for mean frequency were: 5.0 ± 0.4 Hz (*n* = 10 neurons/5 mice) at P14–20, 8.5 ± 0.5 Hz (*n* = 13 neurons/9 mice) at P22–28, 7.8 ± 0.5 Hz (12 neurons/6 mice) at P30–45 and 10.5 ± 0.4 Hz (*n* = 16 neurons/7 mice) at P50–90. **(B)** Mean amplitude of GABA-sIPSCs is augmented in P14–20 HRM compared to aged-matched wild-type (31.6 ± 1.9 pA, *n* = 12 cells/6 mice HRM) and reduced in adult HRM compared to adult wild-type (30.4 ± 1.9 pA, *n* = 17 cells/7 mice HRM). At P22–28 and P30–45, mean amplitude was similar between both genotypes (37.8 ± 2.8 pA, *n* = 15 neurons/8 mice HRM Juv; 36.7 ± 3.5 pA, *n* = 16 neurons/7 mice HRM Ado). *F*_(7,103)_ = 7.421, *P* < 0.0001, ANOVA. Mean frequency of GABA-sIPSCs is augmented in P14–20 HRM compared to aged-matched wild-type (8.1 ± 0.4 Hz, *n* = 12 cells/6 mice HRM) and reduced in adult HRM compared to adult wild-type (6.9 ± 0.5 Hz, *n* = 17 cells/7 mice HRM). In juvenile and adolescent, mean frequency was similar between both genotypes (9.0 ± 0.6 Hz, *n* = 15 neurons/8 mice HRM Juv; 8.0 ± 0.5 Hz, *n* = 16 neurons/7 mice HRM Ado). *F*_(7,103)_ = 9.499, *P* < 0.0001, ANOVA. **(C)** Representative traces of GABA-sIPSCs recorded at -70 mV from both genotypes at indicated developmental stages. Calibration: 50 pA, 200 ms. **(D)** Left: decay time constant of GABA-sIPSCs during maturation of wild-type mice. Values were: 12.3 ± 0.5 ms (*n* = 10 neurons/5 mice) at P14–20, 10.4 ± 0.5 ms (*n* = 13 neurons/9 mice) at P22–28, 9.9 ± 0.3 ms (*n* = 12 neurons/6 mice) at P30–45 and 9.0 ± 0.3 ms (*n* = 16 neurons/7 mice) at P50–90. Right: decay time constant is slower in adult HRM compared to aged-matched wild-type (11.0 ± 0.3 ms, *n* = 17 cells/7 mice HRM) and was not different between both genotypes from pre-weaning to adolescent period. *F*_(7,103)_ = 6.212, *P* < 0.0001, ANOVA. **(E)** Representative normalized traces illustrating decay acceleration of sIPSCs during maturation in wild-type (top) and slower decay in adult HRM compared to age-matched wild-type. Calibration: 10 ms. **(A,B,D)** Data are expressed as mean ± SEM. ^∗^*P* < 0.05, ^∗∗^*P* < 0.01, ^∗∗∗^*P* < 0.001, ANOVA.

Spontaneous GABA_A_-mediated inhibitory post-synaptic currents (sIPSCs) were recorded in layer 5/6 PrPFC pyramidal neurons in whole-cell configuration (**Figure 2**). In wild-type mice, both mean amplitude and frequency increased between P14 and P90 (*F*_(3,47)_ = 14.39, *P* < 0.0001 and *F*_(3,47)_ = 24.53, *P* < 0.0001 respectively, ANOVA; **Figure 2A**). In contrast, both parameters remained similar throughout maturation in HRM (*F*_(3,56)_ = 1.943, *P* = 0.1331, ANOVA mean amplitude and *F*_(3,56)_ = 3.515, *P* = 0.3188, ANOVA mean frequency; **Figure 2B**). When compared between genotypes, mean amplitude and frequency were higher in pre-weaning HRM, similar during the juvenile and the adolescent periods and reduced in adult HRM (**Figures 2B,C**). These results indicate that synaptic activity at inhibitory synapses increases with maturation in wild-type layer 5/6 PrPFC whereas it does not change during maturation of HRM.

During brain maturation, the subunit composition of GABA_A_ receptors undergoes changes from predominantly containing α2 to α1 subunit, thus contributing to faster kinetics observed with age (Dunning et al., 1999; Davis et al., 2000; Eyre et al., 2012; Ehrlich et al., 2013). We next examined whether GABA-sIPSCs kinetics from layer 5/6 PrPFC pyramidal neurons displayed maturation-dependent changes. In wild-type, the decay time constant exhibited a large decrease form the pre-weaning period to adulthood (*F*_(3,47)_ = 9.763, *P* < 0.0001, ANOVA; **Figures 2D,E**) showing that GABA-sIPSCs became faster with age. In contrast, the decay time constant remained similar between pre-weaning, adolescent and adult HRM (**Figure 2D**). Of note, the decay time constant was higher in adult HRM compared to age-matched wild-type (**Figures 2D,E**), showing that in HRM GABA-sIPSCs remained in an immature stage.

### Maturation of Short-Term GABAergic Plasticity in Layer 5/6 PrPFC

**FIGURE 3:**
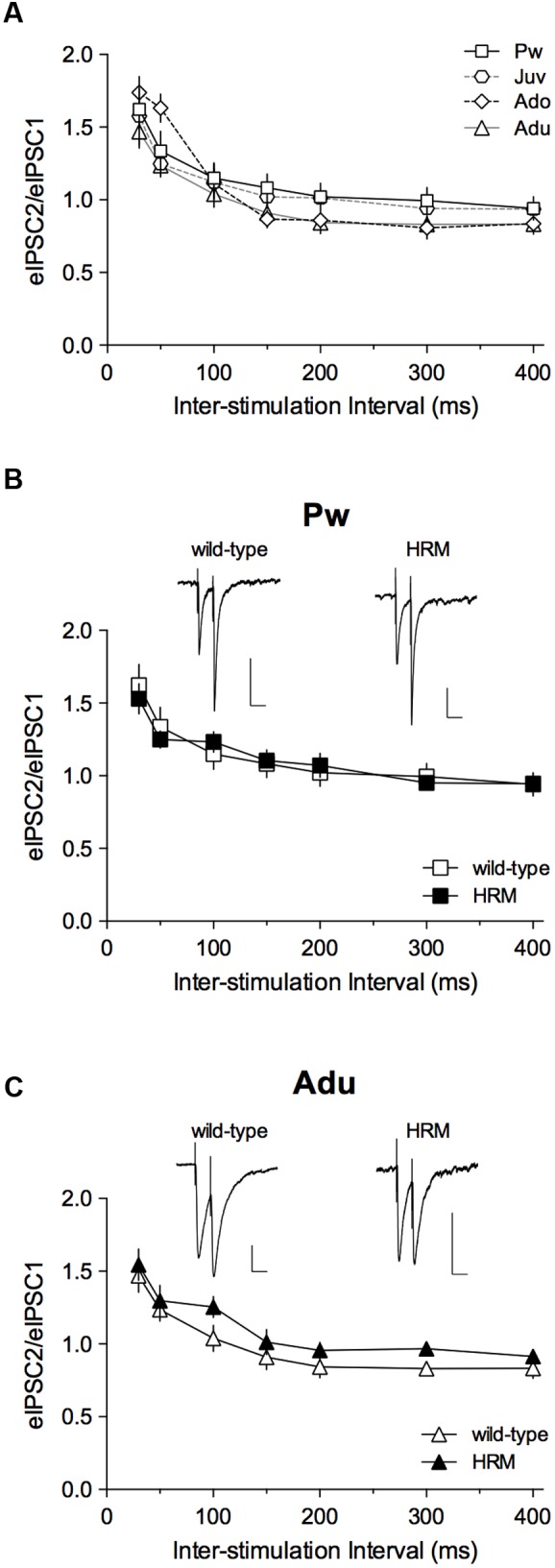
**Maturational profile of short-term plasticity.** **(A)** Paired-pulse ratios (PPRs) of evoked GABA-IPSCs in wild-type mice indicate no difference from pre-weaning to adulthood. Two-way ANOVA (age × inter-stimulation interval) revealed a significant effect of inter-stimulation interval (*F*_(6,186)_ = 61.80, *P* < 0.001), a non-significant effect of age (*F*_(3,31)_ = 0.6857, *P* = 0.5676) and a non-significant interaction between factors (*F*_(18,186)_ = 1.423, *P* = 0.1248). Pw: *n* = 11 cells/5 mice; Juv: *n* = 7 cells/4 mice; Ado: *n* = 8 cells/4 mice; Adu: *n* = 9 neurons/4 mice. **(B,C)** PPRs of eIPSCs show no difference between wild-type and HRM in the pre-weaning **(B)** and adult **(C)** periods. At both ages, two-way ANOVA (genotype × inter-stimulation interval) revealed a significant effect of inter-stimulation interval (**B**: *F*_(6,96)_ = 25.40, *P* < 0.001; **C**: *F*_(6,96)_ = 32.28, *P* < 0.001), a non-significant effect of genotype (**B**: *F*_(1,16)_ = 5.6 × 10^-3^, *P* = 0.9411; **C**: *F*_(1,16)_ = 1.461, *P* = 0.2444) and a non-significant interaction between factors (**B**: *F*_(6,96)_ = 0.6067, *P* = 0.7244; **C**: *F*_(6,96)_ = 0.2614, *P* = 0.9534). HRM: *n* = 7 neurons/4 mice Pw **(B)** and *n* = 9 neurons/6 mice Adu **(C)**. Representative recordings of 50 ms interval evoked GABA-IPSCs in pre-weaning wild-type and HRM (**B**; calibration: 100 pA, 50 ms) and in adult wild-type and HRM (**C**; calibration: 50 pA, 50 ms). Data are expressed as mean ± SEM.

To test whether this altered pattern in pre-weaning and adult HRM resulted in modification of short-term plasticity, we analyzed the PPR of evoked IPSCs (eIPSCs; **Figure 3**) during maturation in wild-type mice (**Figure 3A**) and then compared PPR between both genotypes during the pre-weaning (**Figure 3B**) and adult periods (**Figure 3C**). This form of plasticity, which depends on release probability was identical in wild-type at all developmental epochs and all intervals tested (**Figure 3A**) and between both genotypes at P14–20 and P50–90 at all intervals tested (**Figures 3B,C**). These results show that decreased reelin levels do not affect short-term plasticity of layer 5/6 pyramidal neurons GABAergic synapses during the first 3 months of PrPFC maturation.

### Reelin Haploinsufficiency Impairs the Developmental Trajectory of the E/I Balance

Alterations in the ratio of excitatory (glutamatergic)/ inhibitory (GABAergic) neurotransmission in the PFC have been proposed to play a role in psychiatric disorders of schizophrenic and ASD patients (Bicks et al., 2015). An altered E/I balance has also been reported in mouse models of several psychiatric disorders (Gandal et al., 2012; Gkogkas et al., 2013; Lee et al., 2015).

**FIGURE 4:**
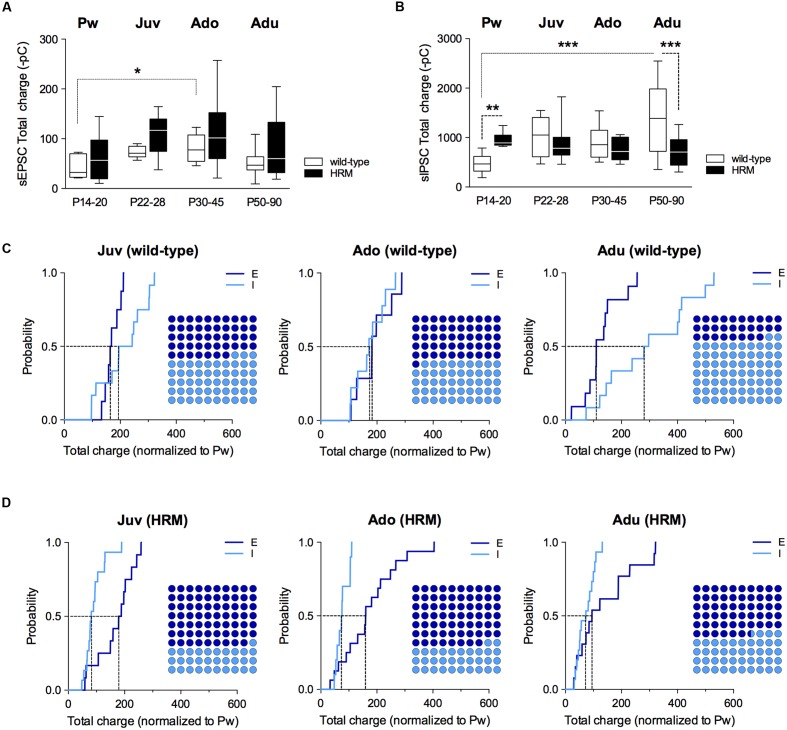
**Maturational profile of the E/I balance in wild-type and reelin-haploinsufficient mice.** **(A)** Box plot showing the interquartile range with whiskers at minimum and maximum data points of the AMPA-sEPSCs total charge measured over a 6 min period in wild-type mice (*n* = 8 neurons/4 mice P14–20, *n* = 8 neurons/6 mice P22–28, *n* = 7 neurons/4 mice P30–45 and *n* = 11 neurons/9 mice P50–90) and HRM (*n* = 11 neurons/6 mice P14–20, *n* = 12 neurons/7 mice P22–28, *n* = 16 neurons/7 mice P30–45 and *n* = 13 neurons/6 mice P50–90). Horizontal lines represent the median sEPSCs total charge. **(B)** Same as A for total charge of GABA-sIPSCs measured over a 6 min period in wild-type mice (Pw: *n* = 9 cells/5 mice, Juv: *n* = 12 cells/8 mice, Ado: *n* = 10 cells/5 mice and Adu: *n* = 12 cells/5 mice) and in HRM (Pw: *n* = 9 cells/5 mice, Juv: *n* = 15 cells/8 mice, Ado: *n* = 10 cells/5 mice and Adu: *n* = 15 cells/7 mice). Error bars represent SEM. ^∗^*p* < 0.05, ^∗∗^*P* < 0.01, ^∗∗∗^*P* < 0.001, Mann–Whitney *t*-test. **(C)** Cumulative frequency distributions of sEPSC (E) and sIPSC (I) total charge transfer obtained for each wild-type neurons within juvenile, adolescent and adult epochs normalized to the mean value of total charge transfer calculated for wild-type P14–20 neurons. E: *n* = 8 Juv, 7 Ado and 11 Adu; I: *n* = 12 Juv, 10 Ado and 12 Adu. Insets: dot plots showing the proportion of E versus I during maturation extrapolated at *P* = 0.5 from the corresponding cumulative distributions. **(D)** Same as C but for HRM. Total charge transfer of each HRM neuron was normalized to the mean value of total charge transfer obtained for pre-weaning HRM. E: *n* = 12 Juv, 16 Ado and 13 Adu; I: *n* = 15 Juv, 10 Ado and 15 Adu. *n* represents the number of neurons.

**FIGURE 5:**
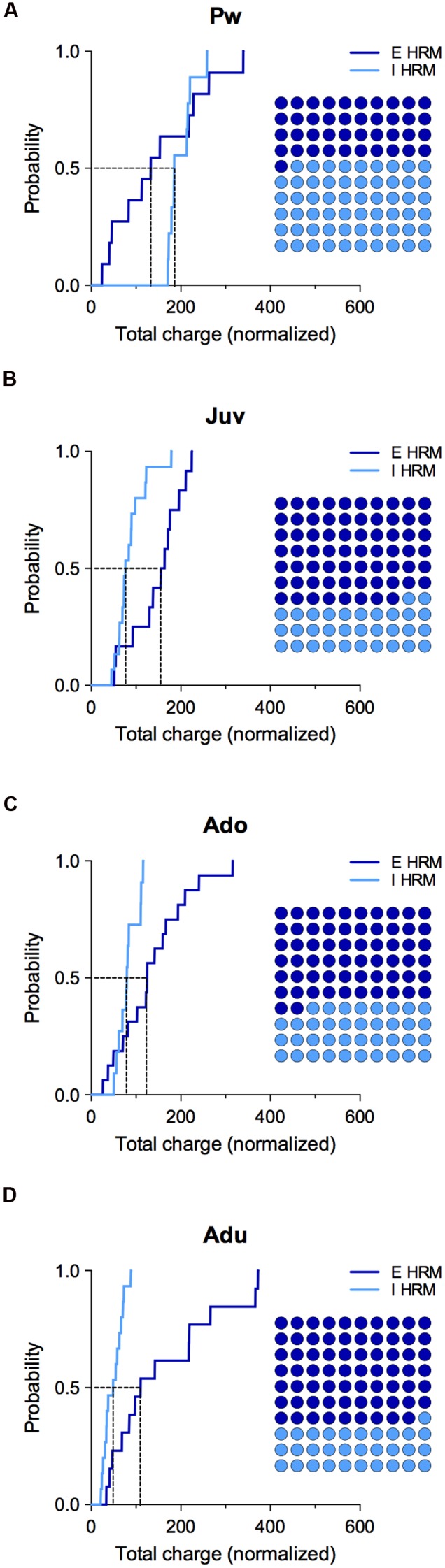
**Effect of reelin haploinsufficiency on the maturation of E/I balance.** **(A–D)** Cumulative frequency distributions of E and I total charge transfer for HRM neurons normalized to the wild-type age-matched mean value are shown at all maturational epoch. Insets: dot plots showing the maturation of the proportion of E versus I extrapolated at *P* = 0.5 from the corresponding cumulative distributions. Pw: E = 11 and I = 9 neurons, Juv: E = 12 and I = 15 neurons, Ado: E = 16 and I = 10 neurons, Adu: E = 13 and I = 15 neurons.

Therefore, we next examined the maturation of the E/I balance (**Figure 4**) and whether it was modified by reelin haploinsufficiency (**Figure 5**). First, we examined the total charge transfer from whole-cell recorded spontaneous AMPA-mediated EPSCs (sEPSCs; **Figure 4A**) and sIPSCs (**Figure 4B**), a parameter which accounts for both frequency and amplitude of spontaneous synaptic events. The total charge transfer of sEPSCs was augmented from pre-weaning to adolescence in wild-type (*F*_(3,30)_ = 4.235, *P* = 0.0131, ANOVA; **Figure 4A**). It was neither significantly different in HRM (*F*_(3,48)_ = 1.845, *P* = 0.1517, ANOVA; **Figure 4A**) nor between the two genotypes within each developmental period (*F*_(7,78)_ = 2.551, *P* = 0.203, ANOVA; **Figure 4A**). These results are in accordance with the maturational profile of AMPA-sEPSCs mean amplitude (Iafrati et al., 2016) and frequency in both wild-type mice and HRM (**Supplementary Figure S2**). The total charge transfer of sIPSCs increased in wild-type adult compared to pre-weaning (*F*_(3,39)_ = 6.913, *P* = 0.0008, ANOVA; **Figure 4B**) as expected from the maturational profile of wild-type sIPSCs mean amplitude and frequency (**Figure 2A**). The sIPSCs total charge transfer was higher in P14–20 HRM and reduced in HRM at P50–90 compared to age-matched wild-type mice whereas it was identical between both genotypes at juvenile and adolescent stages (*F*_(7,84)_ = 5.836, *P* < 0.0001, ANOVA; **Figure 4B**).

Subsequently, we computed the relative changes in excitation and inhibition using normalized total charge transfer from sEPSCs and sIPSCs (**Figures 4C,D** and **5**; Gkogkas et al., 2013). We first analyzed the maturation of E/I balance within each genotype (**Figures 4C,D**). In wild-type, we found that the normalized total charge transfer from sEPSCs and sIPSCs was similar between P22 and P45 showing that the E/I balance did not vary during juvenile and adolescent stages (**Figure 4C**, Juv and Ado), and that E and I were represented in equal proportion (**Figure 4C**, Dot plots). However, at P50–90 the normalized sIPSCs total charge transfer was significantly increased compared to juvenile and adolescent as shown by the right-shift in the sIPSC cumulative distribution (**Figure 4C**, Adu), indicating that the E/I balance dramatically shifted to an increased proportion of I at adulthood. In HRM, the E/I balance remained identical from juvenile to adolescent stage and was characterized by a higher proportion of E as shown by a right-shift in the normalized sEPSC total charge transfer cumulative distributions compared to age-matched sIPSC cumulative distributions (**Figure 4D**, Juv and Ado). In adult HRM, the normalized sEPSC total charge transfer cumulative distribution shifted to the left compared to juvenile and adolescent, resulting in an equal proportion of E versus I that was comparable to the E/I balance observed in juvenile and adolescent wild-type (**Figure 4D**, Adu). These data show that in wild-type PrPFC the postnatal maturation of the E/I balance is characterized by a shift toward increased I during adulthood. In contrast, in HRM the E/I balance did not dramatically change during the same developmental epochs and remained in an immature stage characterized by a larger proportion of E versus I.

To evaluate whether reelin-haploinsufficiency altered the maturation of the E/I balance, we compared the E/I balance between both genotypes at all developmental epochs (**Figure 5**). During the pre-weaning period, the normalized total charge transfer in HRM relative to wild-type mice was larger for sIPSCs as indicated by the right-shift of I cumulative distribution (**Figure 5A**), indicating an increased proportion of I in the E/I balance of pre-weaning HRM compared to age-matched wild-type. At the juvenile stage, the E/I balance in HRM switched to the opposite direction and displayed a reduced proportion of I compared to wild-type as shown by the left shift in the cumulative distribution of total sIPSC charge transfer relative to wild-type mice (**Figure 5B**). The decrease in the proportion of I persisted throughout adolescence and adulthood (**Figures 5C,D**). Therefore, these results show that the maturational sequence of the E/I balance of the PrPFC is disrupted by reelin-haploinsufficiency.

## Discussion

This study describes for the first time analysis of the impact of reelin haploinsufficiency on multiple GABAergic parameters during postnatal maturation (2 weeks to 3 months) of deep layer PrPFC pyramidal neurons. Namely, we investigated alterations in the polarity of GABA action, postnatal maturation of GABAergic synaptic inputs and the developmental sequence of the E/I balance in both wild-type and HRM mice.

In accordance with studies performed in other developing brain structures (Ben-Ari et al., 2007; Kirmse et al., 2015), we found that after 2 weeks of postnatal development GABA exhibits an inhibitory action in PrPFC deep layers. It remains to be determined whether the time course of the developmental excitatory-inhibitory GABA sequence in the PrPFC is identical to other brain structures.

We provide the first evidence that GABAergic synaptic transmission undergoes significant changes during PrPFC postnatal development. In wild-type mice, we show a maturation of the function of GABAergic synapses on pyramidal layer 5/6 PFC neurons with an increase of transmission efficacy with age which reached maturity at ∼2–3 months of age. Similar results have been described in primate dorsolateral PFC (Gonzalez-Burgos et al., 2015). Specifically, we found that both the amplitude and frequency of GABA_A_-mediated sIPSCs increased between P14 and P90 whereas the PPR did not change, suggesting a post-synaptic locus of developmental alteration. These changes could result from the functional maturation of the expression of GABA_A_ receptor subunits (Fritschy and Panzanelli, 2014) and/or from an increase in the number of post-synaptic GABAergic sites as in the rat frontal cortex, where a transient increase in the expression of gephyrin, the post-synaptic scaffolding protein that anchors GABA_A_ receptors has been reported around P21–25 (Pinto et al., 2013). Similar to changes reported in other developing brain areas (Hollrigel and Soltesz, 1997; Dunning et al., 1999; Ehrlich et al., 2013), we found changes in spontaneous GABA_A_-mediated IPSC kinetics with slow IPSCs in early period followed by a sharp reduction of the decay time constant from juvenile to adulthood. It remains to be determined whether the maturation of IPSC kinetics in pyramidal layer 5/6 PFC neurons results from changes in expression of GABAa receptors subunits (Hollrigel and Soltesz, 1997; Dunning et al., 1999; Ehrlich et al., 2013) or other mechanisms (Draguhn and Heinemann, 1996).

In contrast, in HRM spontaneous GABAergic synaptic transmission remained stable during the same developmental period and displayed an immature phenotype similar to juvenile and adolescent wild-type. Whether GABAergic synaptic inputs on layer 5/6 pyramidal neurons reach their maturity before P14 or after P90 in HRM requires further investigation. The differences in the time course of maturation of GABAergic synaptic transmission in HRM and wild-type littermates resulted in an increased transmission efficacy in P14–20 HRM and a reduction at adult stages compared to age-matched wild-type mice. The latter has been similarly reported in CA1 pyramidal neurons of adult HRM (Qiu et al., 2006).

The E-I balance has been shown to shift during early development in the sensory cortex (Dorrn et al., 2010) and to be a trigger for the onset of critical periods in the developing cortex (Hensch and Fagiolini, 2005). Biochemical measurements support the finding that the E/I balance reaches maturity later in the frontal cortex compared to visual and somatosensory cortices (Pinto et al., 2013). Of particular interest, alterations of the E/I balance have been found in animal models of psychiatric disorders (Gatto and Broadie, 2010) and it was shown that direct alteration of the E/I balance within the PFC has a strong effect on social motivation in mice (Yizhar et al., 2011). Thus, we found it crucial to examine the sequence of the E/I balance during periods of development. We observed that in PrPFC the E/I balance reaches maturity during adulthood and is characterized by a shift toward increased inhibition. In contrast, in HRM the E/I balance did not shift and remained in an immature stage. We also showed that reelin-haploinsufficiency blocked the maturational shift of the E/I balance which occurs during adulthood in wild-type PrPFC. Our findings indicate that the developmental trajectory of the E/I balance is disrupted in HRM, which could prove deleterious for the proper initiation of intense periods of plasticity in the PFC. In turn, this aberrant development may increase vulnerability to PFC-related disorders.

The network of layer 5/6 pyramidal neurons consists of local connections with principal neurons and different types of GABAergic neurons present in the different layers in addition to long-range thalamic imputs. The effect of reelin-haploinsufficiency on local interconnectivity as well as long-range thalamocortical connectivity is unknown. Aberrant thalamo-cortical circuitry has been reported in homozygous reeler mice (Li et al., 2005) as well as modification in GABAergic markers in HRM (Nullmeier et al., 2011). Thus our findings could result from a direct mechanism such as changes in GABAergic local connectivity, may be secondary to a general disruption in cortical development, or result from homeostatic mechanisms involving reduced thalamic input dependent excitation which in turn could cause reduction in GABAergic terminals. Apical dendritic activity of layer 5 somatosensory pyramidal cells is highly sensitive to inhibition mediated by interneurons, presumably Martinotti cells, present in deep cortical layers (Murayama et al., 2009). Of note, some deep cortical Martinotti cells express reelin (Pesold et al., 1999) and constitute one of the main sources of secreted reelin in neocortical superficial layers (Ramos-Moreno and Clasca, 2014). Thus, GABAergic impairment and disruption of the E/I balance observed in HRM could have deleterious effects on the function of cortical circuits such as the dendritic filtering of inputs and encoding of stimuli and ultimately in the processing of information and behavioral adaptation (Barr et al., 2008; Teixeira et al., 2011; Labouesse et al., 2016).

Together, our data show that reelin is necessary for the fine-tuning of GABAergic connectivity and of the physiological E/I balance in the maturing PrPFC. Furthermore, these data indicate that a disrupted developmental trajectory of prefrontal GABAergic microcircuitry leads to an altered E/I balance. Combined with previous findings illustrating disrupted E/I balance in psychiatric disorders, it therefore follows that this aberrant maturation may ultimately manifest as behavioral deficits. While further research dissecting if reelin-haloinsufficiency affects a specific class of PFC interneurons is crucial for concluding the exact nature and mechanisms underlying such consequences, these data lay the groundwork for novel investigations into the mechanistic underpinnings of complex psychiatric diseases which manifest during development.

## Ethics Statement

All experiments were performed according to INSERM ethic rules. This study and protocols were approved by the ethic committee of Marseille under the reference n°2015121715284829-V1n°#3279.

## Author Contributions

LB performed electrophysiology related to **Figures 2** to **5**, conducted the data analysis and contributed to the design of the experiments and to the writing of the manuscript. AS performed experiments related to **Figure 1**, conducted the data analysis and experimental design and contributed to the writing of the manuscript. OL, JI, and AT performed electrophysiology experiments. PC designed the experiments, conducted data analysis, supervised the entire project and wrote the manuscript.

## Conflict of Interest Statement

The authors declare that the research was conducted in the absence of any commercial or financial relationships that could be construed as a potential conflict of interest.
