# Supplementary material for: Reelin-Haploinsufficiency Disrupts the Developmental Trajectory of the E/I Balance in the Prefrontal Cortex
Source: Front Cell Neurosci. 2017 Jan 12;10:308. doi: 10.3389/fncel.2016.00308 (PMC5226963; doi:10.3389/fncel.2016.00308)
Supplement: Supplementary file 1 [file Data_Sheet_1.pdf]

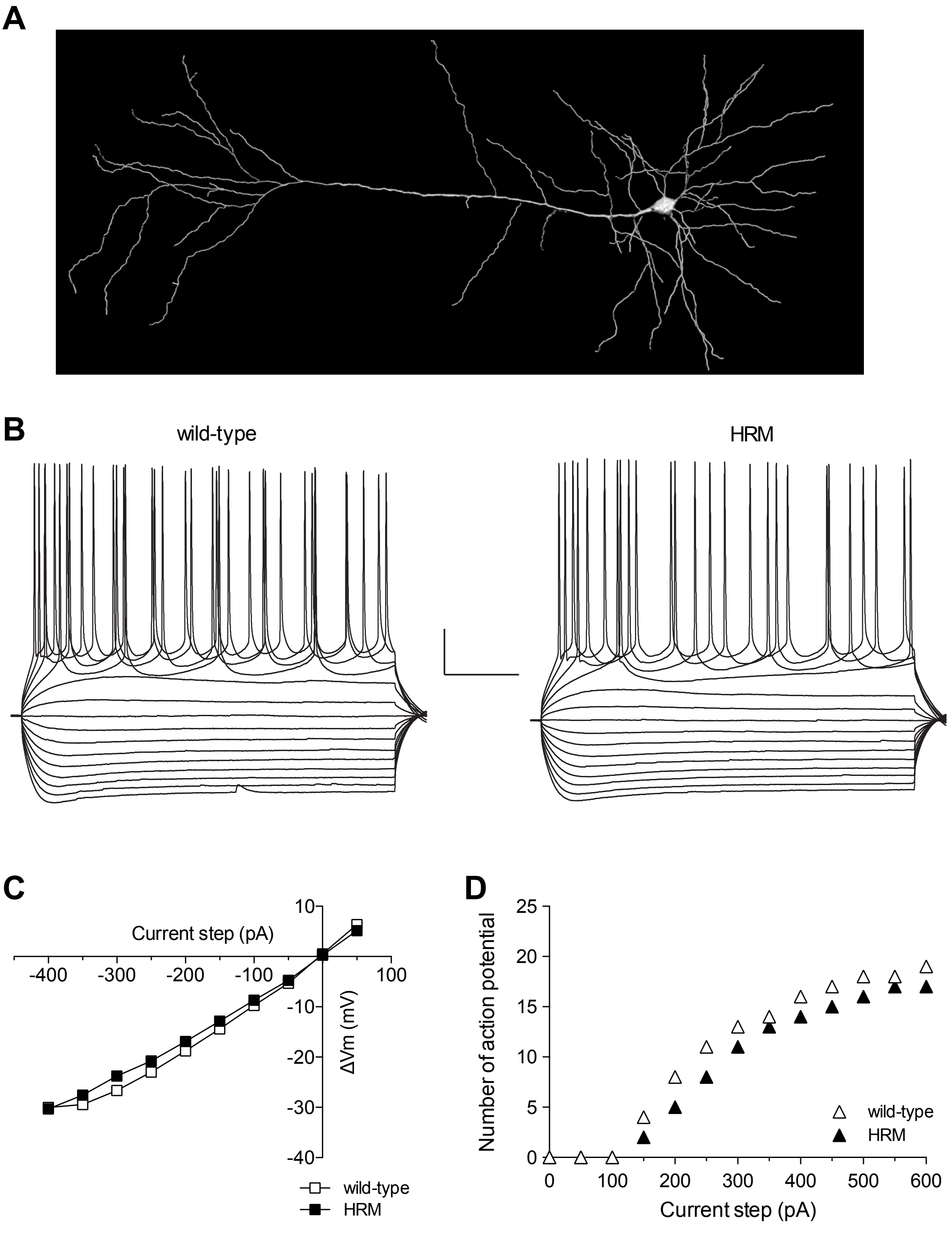

Supplementary Figure 1

Supplementary Figure 1: Intrinsic properties of layer 5/6 PrPFC pyramidal neurons in wild-type and HRM neurons.

(A) Tri-dimensional reconstructed confocal image of layer 5 PrPFC pyramidal neuron filled with neurobiotin during whole-cell recording. Staining and reconstruction were performed as previously described (Iafrati et al. 2014).

(B) Typical membrane responses to somatic current step recorded from wild-type and HRM layer 5/6 PrPFC pyramidal neurons (calibration: 100 mV, 20 ms).

(C) Current-voltage curves corresponding to the two neurons illustrated in **B**. Changes in membrane potential in response to hyperpolarizing and depolarizing current steps.

(D) Current-firing curves indicating the number of evoked action potentials in response to somatic current steps for the two neurons showed in **B**.

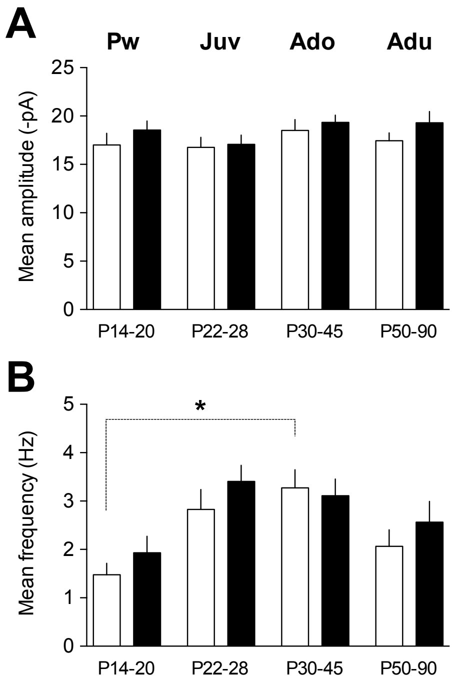

Supplementary Figure 2

Supplementary Figure 2: Properties of AMPA-sEPSCs during maturation.

(A) Average amplitudes were similar during maturation and between genotypes ($F_{(7,78)}$=1.158, *P*=0.3366, ANOVA). Values were in Pw: 17.0 ± 1.2 pA, n=8 cells/4 mice wild-type and 18.5 ± 1.0 pA, n=11/6 mice HRM; Juv: 16.8 ± 1.0 pA, n=8/6 mice wild-type and 17.1 ± 1.0 pA, n=12/7 mice HRM; Ado: 18.5 ± 1.1 pA, n=7/4 mice wild-type and 19.3 ± 0.8 pA, n=16/7 mice HRM; Adu: 17.5 ± 0.8 pA, n=11/9 mice wild-type and 19.3 ± 1.2, n=13/6 mice HRM.

(B) The mean frequency increased in wildtype between pre-weaning and adolescent period (1.5 ± 0.2 Hz at P14-20, 2.8 ± 0.4 Hz at P22-28, 3.3 ± 0.4 Hz at P30-45 and 2.1 ± 0.3 Hz at P50-90; $F_{(3,31)}$=4.503, *P*=0.0098, ANOVA) whereas it was not different in HRM during maturation (1.9 ± 0.3 Hz at P14-20, 3.4 ± 0.3 Hz at P22-28, 3.1 ± 0.3 Hz at P30-45 and 2.6 ± 0.4 Hz at P50-90; $F_{(3,48)}$=2.830, *P*=0.0482, ANOVA).

Error bars represent SEM. * *P* <0.05.
